# Solidarity during the COVID-19 pandemic: evidence from a nine-country interview study in Europe

**DOI:** 10.1136/medhum-2022-012536

**Published:** 2023-12-19

**Authors:** Katharina Kieslich, Amelia Fiske, Marie Gaille, Ilaria Galasso, Susi Geiger, Nora Hangel, Ruth Horn, Marjolein Lanzing, Sébastien Libert, Elisa Lievevrouw, Federica Lucivero, Luca Marelli, Barbara Prainsack, Franziska Schönweitz, Tamar Sharon, Wanda Spahl, Ine Van Hoyweghen, Bettina M. Zimmermann

**Affiliations:** 1 Centre for the Study of Contemporary Solidarity, Department of Political Science, University of Vienna, Vienna, Austria; 2 Institute of History and Ethics in Medicine, Technical University of Munich, Munich, Germany; 3 Institut des sciences humaines et sociales, CNRS, Paris, France; 4 SPHERE ( Sciences, Philosophie, Histoire), CNRS/ Université de Paris/ Université Paris 1 Panthéon-Sorbonne, Paris, France; 5 College of Business, University College Dublin, Dublin, Ireland; 6 Leibniz Center for Science and Society, Leibniz University Hannover, Hannover, Germany; 7 Ethox Centre, Nuffield Department of Population Health, University of Oxford, Oxford, UK; 8 Institute for Ethics and History of Health in Society, University of Augsburg, Augsburg, Germany; 9 Faculty of Humanities, University of Amsterdam, Amsterdam, The Netherlands; 10 Division of Psychiatry, Faculty of Brain Sciences, University College London, London, UK; 11 Life Sciences & Society Lab, Centre for Sociological Research, KU Leuven, Leuven, Belgium; 12 Department of Medical Biotechnology and Translational Medicine, University of Milan, Milan, Italy; 13 Faculty of Philosophy, Theology and Religious Studies and Interdisciplinary Hub for Digitalisation and Society, Radboud University, Nijmegen, The Netherlands; 14 Institute for Biomedical Ethics, University of Basel, Basel, Switzerland

## Abstract

Calls for solidarity have been an ubiquitous feature in the response to the COVID-19 pandemic. However, we know little about how people have thought of and practised solidarity in their everyday lives since the beginning of the pandemic. What role does solidarity play in people’s lives, how does it relate to COVID-19 public health measures and how has it changed in different phases of the pandemic? Situated within the medical humanities at the intersection of philosophy, bioethics, social sciences and policy studies, this article explores how the practice-based understanding of solidarity formulated by Prainsack and Buyx helps shed light on these questions. Drawing on 643 qualitative interviews carried out in two phases (April–May 2020 and October 2020) in nine European countries (Austria, Belgium, France, Germany, Ireland, Italy, The Netherlands, German-speaking Switzerland and the UK), the data show that interpersonal acts of solidarity are important, but that they are not sustainable without consistent support at the institutional level. As the pandemic progressed, respondents expressed a longing for more institutionalised forms of solidarity. We argue that the medical humanities have much to gain from directing their attention to individual health issues, and to collective experiences of health or illness. The analysis of experiences through a collective lens such as solidarity offers unique insights to understandings of the individual and the collective. We propose three essential advances for research in the medical humanities that can help uncover collective experiences of disease and health crises: (1) an empirical and practice-oriented approach alongside more normative approaches; (2) the confidence to make recommendations for practice and policymaking and (3) the pursuit of cross-national and multidisciplinary research collaborations.

## Introduction

Calls for solidarity have been an ubiquitous feature of policymaking since the onset of the COVID-19 pandemic. In many countries, and even at a transnational level, solidarity has been used in appeals to the public to adhere to measures to contain the spread of the virus, as well as a rhetorical device to underline the message that “we are all in this together” (Guttman and Lev 2021). WHO (2020) has embraced the notion of solidarity to underline the need for international cooperation and for knowledge sharing. Solidarity has also been employed as an anchor point to underline the importance of specific policy measures (eg, Hangel et al. 2022; Iftekhar et al. 2021), such as steps to increase vaccine uptake to combat vaccine hesitancy (Prats-Monné 2018). However, we know little about how people have thought of, and practised, solidarity in their everyday lives since the beginning of the pandemic. What role does solidarity play in pandemic times and how does it relate to public health and other measures introduced since the onset of the pandemic?

We build on a practice-based approach to solidarity to answer these questions. We present insights from a large-scale, international, qualitative study that signals a societal longing for institutional transformation—in the form of what we call institutionalised or tier 3 solidarity—to address the many social injustices brought to light and exacerbated by the pandemic, and to show how these insights are relevant for policymaking in pandemic times. Our analysis leads us to reflect on the role of medical humanities in pandemic times: What lessons can we draw for policymaking, healthcare practice and the potential for medical humanities to guide insights into future health crises?

These are important questions to address, especially for a large and multidisciplinary field such as the medical humanities (League of European Research Universities (LERU) 2012). The analysis of solidarity-related practices can help in exploring some salient social justice issues that the pandemic has brought to the forefront of scholarly, public health, political and societal attention, and thereby contribute to one of the longstanding aims of the medical humanities to uncover contexts and meanings of social (in)justice in medical practice and healthcare systems. While the disciplinary, conceptual or methodological focus of the medical humanities can vary depending on how inclusive an approach is taken towards the humanities and social sciences, the patient’s experience is at the centre of most of the work in the field. The medical humanities have thus been crucial in influencing and shaping the idea that patients, and their experiences, should be the core concern of the medical practice and research community. More widely, the humanities and social sciences have long highlighted the fact that societies are built on social relationships at different levels, and that we need to understand these relationships in order to inform policy and healthcare practice (Green and Cladi 2020; Pickersgill and Smith 2021). In our research, we build on the idea that one cannot aim to resolve complex problems or improve healthcare practice without understanding what patients and individual members of society experience. Sadly, a pandemic means that most people will either become a patient, have been a patient or know someone who is or has been a patient. Everyone is affected by a pandemic in one way or another.

Drawing on 643 in-depth, qualitative interviews conducted during two phases (April and May 2020; October 2020) in nine European countries, this article explores solidaristic practices and how these changed between the two periods of the longitudinal study. In line with Prainsack and Buyx (2017), we understand solidarity as practices by which people support others with whom they see themselves as connected in some relevant way— may it be a common goal, a shared identity or the joint fight for a common cause or a social good. In the interviews, we found that appeals to solidarity resonated with people at the beginning of the pandemic, who largely followed public health advice and stayed home during the first lockdowns.

We distinguish solidaristic practices from more rhetorical expressions of solidarity and show how an approach that focuses on concrete actions, inaction or motivations of people to engage in ways that foster or hinder solidarity draws a nuanced picture. It demonstrates where, how (and sometimes also why) different forms of solidarity take place, and where solidarity is seen as lacking. The approach also allows us to make sense of a crucial change that we identified between the two interview phases of the study: whereas acts and expressions of personto-person solidarity were strong in the first interview phase in April and May 2020, in October 2020 they were marked by a longing for more institutionalised forms of solidarity. This may be a common trajectory in times of crisis where a strong sense of solidarity and the need for mutual support decrease over time as the circumstances of the crisis are more normalised and a certain crisis fatigue sets in (Rakopoulos 2016). Beyond this, however, we see it also a result of injustices and inequalities exacerbated, or brought on, by pandemic policies. A practice-based understanding of solidarity allows us to go beyond interpretations of the waning of solidarity as ‘pandemic fatigue’, and instead to underline the need for solidaristic practices to be fostered by public institutions in order to sustain them throughout and beyond crises.

### Background: solidarity in European healthcare, public health and the medical humanities

Solidarity plays a role in healthcare and public health in at least three important ways. First, solidarity is often labelled a core value on which publicly funded healthcare systems in Europe are built. This applies to both tax-funded health systems as well as systems funded through social health insurance contributions. In the case of the former, Saltman (2015, 1) argues that the notion of equity as a guiding principle for how resources are distributed fairly between patients is the ‘conceptual near-equivalent’ of solidarity in social health insurance systems. The underlying idea is that healthcare should be provided and accessible to all as needed, when needed, without negative financial repercussions and in a reciprocal fashion.

Over time, through the institutionalisation of public healthcare, this has led to people accepting the costs of paying into collective pots of healthcare even if they are healthy, knowing that one day when they might need healthcare, they can access it without this having negative consequences for their personal financial situation. This institutionalised form of solidarity hinges on the assumption that all members of society share a vulnerability to ill health, which can be mitigated by a system of mutual support. Against this background, it is easy to see how a health crisis such as the COVID-19 pandemic has the potential to threaten the very foundation on which public healthcare systems rest as practitioners are faced with morally intractable decisions of having to prioritise some patients over others even though the value of mutual support and equal treatment enshrined in the system would suggest otherwise.

Second, solidarity is one of several principles that health practitioners and policymakers might draw on when developing public health guidance in pandemic and non-pandemic times. In the literature on public health ethics and bioethics, solidarity is often juxtaposed or compared with other, more individualised principles that justify public health action, such as autonomy or personal responsibility. The latter notions tend to take centre stage in public health discussions, leading Dawson and Jennings (2012) to argue that solidarity has been an overlooked concept in public health ethics and pandemic ethics frameworks. They conclude that solidarity can and should be applied in a much more explicit way when justifying public health interventions. At the heart of their argument lies an understanding of solidarity as a collective concept, one that transcends many of the other elements of public health ethics such as beneficence, non-maleficence, justice and autonomy (Beauchamp and Childress 2013). For public health interventions to be successful, an understanding is needed that safeguarding values and rights at the individual level, for example the value of autonomy, is not possible without first securing values at the collective level, for example through increasing vaccination uptake. From this point of view, the encouragement of populations to get vaccinated is more than a rhetorical frame, it is a policy instrument deeply rooted in notions of solidarity and humanism.

Third, despite its fundamental importance in healthcare and public health, solidarity remains an opaque concept that is often used but rarely defined (Saltman 2015). This article highlights the value of understanding solidarity through a prism of a practicebased approach that allows us to identify multiple and varied forms of solidarity at different stages of the pandemic, hence circumventing some of the operational problems that might otherwise arise from discussing an often poorly defined concept. We do this by adopting the definition of solidarity by Prainsack and Buyx (2017) that encompasses practice-based understandings, which has proved to be a fruitful lens for researching enactments of solidarity in healthcare (Komparic et al. 2019).

Prainsack and Buyx (2017, 52) argue that the recognition of similarity with another person that in turn gives rise to a solidaristic action is a key element in explaining solidaristic practices: ‘Solidarity is an enacted commitment to carry “costs” (financial, emotional or otherwise) to assist others with whom a person or persons recognise similarity in a relevant respect’. One of the key elements of this definition of solidarity is that it has to be *enacted*, that is to say a concrete action has to emerge from which we can extrapolate that solidarity is being practised.

This working definition of solidarity allows us to analyse interview data from several angles when investigating which actions and practices might be identified as solidaristic practices. In addition to the motivations that people describe as factors for following public health advice or in supporting others during the pandemic, we can reflect on the types of costs people are willing to incur to support others or the way in which they describe groups with whom they feel connected in some relevant way. Importantly for the findings presented in this paper, solidarity can manifest itself at various levels (‘tiers’) (Prainsack and Buyx 2017): the interpersonal level, group level and at the level of institutions and norms. Solidarity at the interpersonal tier refers to practices by which people support others based on shared experiences at the person-to-person level. At this tier, we observe and identify many different forms of solidarity and acts of support between individuals, especially in the first phase of the pandemic.

The second tier refers to manifestations of a shared commitment at a group level, for example, within a self-help group. In our interviews, this emerged in terms of supporting initiatives implemented by new and pre-existing groups (neighbourhood WhatsApp groups, parish, sport clubs). The third tier refers to institutions, policies and other formalised processes that are organised according to the principle of solidarity or that help to realise it (Prainsack and Buyx 2017). The understanding of mutuality and equity in access that underlines many European healthcare systems is probably the most prominent example of such institutionalised forms of solidarity in healthcare. As our findings show, however, that such institutionalisations cannot be taken for granted. Many respondents expressed their frustrations with governments not doing more to institutionalise and support the solidaristic practices that were seen in great abundance at the interpersonal level of solidarity.

The concept of solidarity is particularly useful in thinking about some of the normative and applied ambitions within the medical humanities as a broad field that draws on methods, concepts and content from different humanities and social sciences disciplines (Shapiro et al. 2009). First, it supports the field’s ambition of uncovering, providing and understanding contexts and experiences in medicine and healthcare (Cole, Carlin, and Carson (2015)). In investigating the experiences of respondents during the pandemic, we found that the practice-based understanding of solidarity provides a useful lens for inter-preting what respondents told us about their motivations, hopes, expectations and fears regarding public health measures.

Second, the concept can offer insights for the medical community generally, and medical students specifically. By investigating concrete practices of solidarity, we gained insight into instances related to public health or the management of the pandemic in which respondents felt that solidarity was lacking, thereby creating opportunities for formulating recommendations for practitioners and policymakers alike. Incidentally, this ties in with a third ambition found in some of the medical humanities literature: the call for more empirical and policy-oriented work (Pickersgill and Hogle 2015). Last but not least, the concept of solidarity plays an increasingly important role in scholarly thinking in the humanities, the social sciences and increasingly in public health ethics and bioethics (eg, Buyx and Prainsack 2018; Dawson and Jennings 2012; Dawson and Verweij 2012; Jennings 2018).

## Methods

This publication has been made possible by the joint work of the Solidarity in Times of a Pandemic (SolPan) research commons, a large, multidisciplinary research consortium set up at the beginning of the pandemic to explore people’s experiences (Zimmermann et al. 2022). SolPan is a large-scale qualitative comparative research study comprising interviews with residents from nine European countries (Austria, Belgium, France, Germany, Ireland, Italy, The Netherlands, German-speaking Switzerland and the UK) (Wagenaar et al. 2022).

Participants were recruited through online advertisement via university websites, social media networks, convenience sampling, snowballing and purposive sampling (Bryman 2016), aiming to cover a range of demographics. Age, gender, income, employment status, education, household situation and rural or urban living area were assessed (see online supplementary table for the demographic distribution of interview participants). All participants received a study information leaflet prior to the interview. Formal consent to participate was obtained orally directly before the interview. Both the consent and the subsequent interview were recorded on a digital recorder or using a video chat recorder compliant with the countries’ data protection regulations.

Even though interviews were held in the participating countries’ official languages, all country teams used the same qualitative interview guide developed by the SolPan research commons (SolPan Consortium 2021). Interviews were conducted with the same participants in April and May 2020 (T1) and October 2020 (T2). Participants were asked about their practices and lived experiences during the COVID-19 pandemic with the aim to assess the reasons behind those practices. Even though solidarity served as the theoretical framework for the study, we did not use this term in the interview guide to avoid socially expected answers. Instead, we asked about how participants protected themselves and others against COVID-19, how they supported others, how they accessed information they trusted, their perceptions on COVID-19-related policies and regulations and future expectations. Participants were encouraged to talk freely about their lived experiences even if answers diverged from the interview topics. Only audio, and no video material, was stored for transcription and transcripts were pseudonymised. Interviews lasted between 25 and 90 min.

For data analysis, the SolPan research commons inductively developed a coding scheme that was applied to all interviews using Atlas.ti or NVivo (SolPan Consortium 2021a). Interview data coded with the codes ‘supporting practices’, ‘solidarity’, and ‘protecting others’ was analysed in-depth by each country team in the interviews’ original language. Using the concept of solidarity proposed by Prainsack and Buyx (2017), country-specific analytic reports in English language were written, including descriptions of solidaristic practices as described in the interviews, the motivations behind those practices, perceived costs, what tier of solidarity practices accounted for as well as references to similarities and differences with individuals or groups people were solidaristic with. Moreover, attention was paid to whether and how these practices changed from April to October 2020. Those reports were then used as a basis to structure and compare findings between countries, which was done first through exploratory meetings among all coauthors and then in small analytic groups including two to three researchers from each country. Findings were then reassessed by each country team for consistency with their data.

Interview excerpts were chosen in collaboration between the country teams. The consortium devised an interview key that ensures pseudonymity but allows readers to identify the country and interview phase from which the excerpt stems, which is used throughout this article. The interview identifier is provided in brackets at the end of every quotation and includes the interview phase (T1 or T2), the country code (AT—Austria, BE—Belgium, CH—Switzerland, DE—Germany, FR—France, IE—Ireland, IT—Italy, NL—The Netherlands, UK—United Kingdom), the initials of the researcher who conducted the interview and the interview number.

### Patient and public involvement

Patients and the public were not involved in the design phase of this study because the research project was set up at the onset of the pandemic in which co-production of research projects was difficult due to lockdowns as well as the speed in which the project needed to be set up from scratch (3 weeks). The interview guide for the first phase of the interviews was conceptualised by members of the SolPan consortium based on the available literature on solidarity, and based on the public discourse on solidarity in different countries. The interview guide for the second phase of interviews was informed in part by the findings of the first phase, and by priorities raised by respondents. The study findings continue to be disseminated to the participants, and to the public, in the form of blog posts, media communications and academic publications. The precondition for this dissemination was that interviewees gave their consent to being informed about the outcome of the study findings at the end of the interview.

### Findings

#### Person-to-person and group-based solidarity

Interviews carried out across Europe at the start of the pandemic suggest the emergence of novel forms of interpersonal and group-based solidarity. We found that the practices composing this kind of solidarity were similar in different European countries. Most prominently they included provision of material and emotional support, which takes the form, typically, of (i) financial aid, (ii) provision of food and basic items to those most in need (funding campaigns), (iii) home delivery of food and other basic items (especially to the elderly) and (iv) ‘social’ support in the form of organising telephone rotas to ensure that people who live alone are not abandoned, setting up neighbourhood WhatsApp groups to organise socially distanced on-street get-together or creating solidaristic hampers where people leave what they want and people in need can take without asking. Moreover, many interview participants described doing the groceries for elderly neighbours or contacting friends and family who live alone more frequently. This type of solidarity is often expressed through a heightened level of attention to the needs of others and the desire to do something for the community.

Across the spectrum of solidaristic practices, several interesting commonalities can be traced. We noticed in data across all countries that many participants talked about their motivations for complying with public health measures to contain the pandemic as rooted in understandings of solidarity. Even if the term ‘solidarity’ was not always used, solidaristic reasonings were displayed in terms of making sacrifices and helping each other as a way out of the crisis. In the words of our interviewees:

Following guidelines is at a cost to your own, it could be at a cost to your own business, it could be at a cost of your ability to interact with your family, with your friends, your social interactions, you know upholding the guidelines comes at a cost, but we all seem to be quite happy to do it, given that we know that the value it has to each other. (T2IEFOK02)

Everyone dutifully does what is required of them. I notice that there is a lot of discipline, much more than you might have thought of beforehand. Of course, there is also a kind of underlying solidarity, a bit of looking out for each other. (T1NLMP06)

We see here not just an abstract sense of solidarity or sense of duty, but a concrete desire to protect others, especially those who are perceived to be at greater risk than our respondents considered themselves to be. This translates into a willingness to accept the costs and sacrifices to personal freedoms that go hand-in-hand with complying with containment policies.

In singling out vulnerable people when sharing thoughts about why measures are adhered to, our data give empirical support to theories about the need to identify people or groups with whom one feels solidaristic in order to give substance to solidarity. Groups that were mentioned in the interviews included front-line healthcare workers, people who are not able to work from home, small business owners and those in precarious working and living conditions such as the unemployed or people with refugee status:

Hopefully this crisis requires a lot of solidarity with older people, with the healthcare workers, with other workers who are most affected by that. (T1IESV04)

It is like this, yes [upon being asked if the interviewee adheres to the measures]. But less out of a concern that I could become sick myself, but merely, if one does not follow it [the measures], yes, it would especially affect the generation above me that would perhaps become infected through me and, yes, it [the older generation] is clearly more in danger than I am at the moment. (T1ATBP10)

So, I am not scared that they [clients of the interviewee] carry something [contagious] for me that could harm me. […] it is really important to me that I don’t give it to anyone who is a risk patient. And that’s why one has to be careful. One never knows with whom one would have contact, if one were infected, before even knowing that one is infected. (T1CHBZ19)

On the other hand, many respondents saw instances of other people’s non-compliance to public health measures as unsolidaristic:

So, we have neighbours, they party with friends every day. Which I don’t find great because I then think, yes, then, firstly we will perhaps still be stuck in these lockdowns for longer when everything [the virus] breaks out again. And it could also be […] that people die, this I find so…it gets on my nerves if people do not at least try to get through this together as much as possible. […] And one day my sister, who no longer works as a nurse […] but she has now been called up to work as a nurse again through a compulsory measure and she had to sign a document to say she will be available up to 60 hours a week. And as she told me this, some friends arrived at my neighbours’ house with bottles of wine and started making noise. And in that moment I was so angry […] because I thought why does my sister maybe then have to care for such idiots, I don’t accept this. But yes, I don’t know, such unsolidaristic behaviour really annoys me. (T1ATBP04)

At the same time, some of our interviewees justified certain infringements of pandemic measures on the basis of solidarity. Some participants broke the rules to support others in need of help socially or psychologically, especially during lockdowns.

I went to visit him [an elderly friend living alone]. One morning I defied the law, because we were in full lockdown, but I was worried because I had not heard from him, he was not replying to the texts on our chat anymore. I knew where he lived, so like a thief in the night, slipping from corner to corner in the street, I reached the neighbourhood where he lived and I saw him in his house. (T3ITIG03)^1^

The neighbours’ son had gone missing, they were completely panicked, and then we took care of their younger child. It is actually not allowed, but you do it anyway, because that panic is more important than […] a guideline […]. What you also do more often because of that Covid is that you take walks in your own village. It’s actually very strange, you never do that otherwise and we do that now. And on such a walk we came across a man who had fallen with his walker, but we helped him up and brought him home. I sometimes make exceptions in that sense. I see that more as a kind of priority rule, you have to do things like that. […] So for me these are not measures that are taboo, but they are measures to limit risks as much as possible Every now and then you have to do something and make an exception, I think. But not in the sense that we secretly have parties or anything, we don’t. (T2NLMP06)

These quotations illustrate the types of reasoning that were employed in instances in which helping others in an emergency situation or during tough times were viewed as more important than the strict adherence to the lockdown rules. People were aware that, strictly speaking, they were not allowed to engage in such practices but they were willing to accept the possible consequences in order to assist others:

We are staying in a student residency and a close friend of ours feels lonely. […] He’s seeing a psychologist as well. And I don’t know, normally we’re not allowed to see him or visit him but we do invite him to dinner every day, just to give him some structure in his day. And yes, to carry his mental pressure or burden a little bit […]. Yes, to me that matters more than leaving him alone in his room, knowing that he has a hard time mentally. (T1BEEL05)

In other words, in several situations the willingness to act in a supportive manner manifested itself in actions with potentially significant costs for the individual, in the form of fines or other reprimands, if one were caught breaking the rules. Such instances illustrate the nuances of solidaristic actions brought to light through a practice-based definition of solidarity. These forms of support occurred primarily during the first lockdown phases of the pandemic, in which rules and guidelines provided little room for exception or justified infringements.

By the time of our second round of interviews, in October 2020, many participants (across all countries) expressed fatigue with the pandemic situation, which corresponded with the public debate in many countries about lower social cohesion and less person-to-person solidarity. Participants also explicitly mentioned that they had not maintained some solidaristic practices that they had engaged in or observed in April 2020 because it was too difficult—for financial, social or psychological reasons. Some related the disappearance of organised solidarity initiatives to the mere fact that they were not as greatly needed as before. Many were confident that supporting initiatives and groups would swing back into action if needed.

Yes, well, that’s decreased now, but for the reason that the older people are now going out again themselves. Not in the old people’s home, someone still does the shopping for my father, but for example for my former bosses, for whom they also did the shopping, they now go out themselves, so they no longer have so much to worry about. (T2DENH03)

When the situation gets tragic again, [these practices] will appear again. (T2ITPC02)

To sum up, solidarity was seen as one of several motivating factors to comply with measures and to engage in practices to support others. Interestingly, regarding instances where rules and measures were seen as inefficient or unfair, not sticking to the rules was sometimes also seen as solidaristic. Non-adherence to rules that were seen as useful, however—such as wearing face masks, or keeping a physical distance to others in crowded places—was explicitly labelled unsolidaristic. Especially in the second phase of the interviews, we saw that fatigue and frustration with government (in)action (see below) can affect people’s willingness, ability and endurance to act in solidarity. This suggests that acts of solidarity also depend on opportunities and circumstances in which solidarity can flourish; the longer a difficult situation lasts, and the more people lose confidence in collective forms of solidarity, the more likely it is that solidarity at the interpersonal level will also be difficult to sustain, and the more likely it will be that people long for a transition to more collective forms of solidarity.

### The longing for collective and institutionalised solidarity

Many participants engaged in reflections about how their personal practices can contribute to safeguarding the healthcare system. Van Hoyweghen and Lievevrouw (2020) show that such reflections were a key motivating factor for compliance with public health measures in Belgium. Interviewees in France also expressed gratitude for what they perceived as the continuation of institutionalised solidarity in healthcare, for example when medical care continued even in the absence of a prescription for a particular intervention:

There’s a lot of mutual aid stuff and also shopping for people who can’t. That’s good. I need injections, the nurse came, I don’t even have a prescription, but she comes anyway. As they’ve closed the practice. It’s small stuff, but it’s good. (T1FRMG04)

In many countries, reflections of one’s role in the collective realm of solidarity, and of the importance of this realm, led to concrete actions, or rather to the refraining from risky actions that might lead to injuries as an example of practice-based forms of solidarity:

[…] More than usual I pay attention to a healthy lifestyle […] so that I do not get ill. I would also not do any risky things right now because, I think to myself, I do not want to have to get treatment in a hospital unnecessarily. Something somebody else perhaps needs, so the space [in the hospital]. (T1ATKK05)

These reflections may be interpreted as signalling an understanding of the healthcare system as deeply solidaristic in nature, something that needs to be supported by individuals (what can I do to protect the system?) and government action (what can the government do to protect the system?) alike. We will return to this argument in our discussion.

In both rounds of interviews, participants also expressed concern and disappointment that there was not more solidarity at regional, national and international level. Many participants in all nine countries were unhappy about the support provided by their governments: for those who had lost work as a result of the pandemic, support was often seen as inadequate. During the first phase, participants were unclear how the supporting process would work: would they hear from someone about their case? When would the payments arrive? How would they make ends meet or pay bills in the meantime? Also, participants complained that support measures were implemented unequally, as some categories like irregular or even independent workers were left out from governmental support initiatives:

In Switzerland, there were [political] debates concerning [supporting] the self-employed, but I’d find it also important to talk about rents, for example. Because those running costs remain, they could have been pushed out or eliminated. (T1CHNH01)

I am not earning anything at the moment. It is true that from a state perspective I was not earning anything before either, but they should also care for those who were not working [prior to Covid-19]. (T1IT-FL08)

Demonstrating the complicated interweaving of person-to-person as well as institutionalised solidarity, one participant argued that in Ireland there is a general lack of institutionalised support, and that it ‘forced’ the existing widespread reciprocal support at individual and group-based level:

We often say in Ireland it is because of the lack of services, we grew out of the lack of services you see that weren’t being provided for people. Unfortunately, sometimes the Government depends on us but at the same time I think it is very nice that all these people who try, you know what I mean, to do something for their neighbour or their town or their you know some little things where you are living to make your environment good. (T2IEIG03)

Participants criticised some expressions of solidarity that were encouraged at the institutional level as merely symbolic, tokenistic and even hypocritical, while they longed for more concrete forms of support. For example, many interviewees across the nine countries reflected on acts of clapping for carers such as frontline healthcare workers in the first weeks of the pandemic—something which was put forward by different media sources as a prime example of the upsurge of solidarity in times of a crisis. Our respondents, however, were rather critical of such expressions of support as ‘acts of solidarity’. It was something that was cited by respondents as creating a sort of ‘community’ feeling or togetherness in a difficult time, but even respondents who participated in these national clapping initiatives did not always perceive them as being a form of solidarity, because of the low to no costs involved in showing this support, and because, in most countries, it did not translate into concrete support at the institutional-political level such as an increase in funding for hospitals and other healthcare facilities.

[…] To stand at the window at 7 or 8 pm and to clap […], well, you can do that. But I think this is not an expression of solidarity necessarily for the people working in the hospitals or in care homes. It is a way, an attempt, that people stay optimistic, so showing optimism. I think this is okay, it makes sense. But I would not say that it is a very strong, solidaristic measure. (T1DEAS03)

No, of course I think that’s a nice sense of solidarity at 8pm. But when I hear it, I think ‘wow they still keep it up’. Even my mother, she’s a nurse, but she doesn’t really think that this attention is necessary either and there was also talk of a pay rise and that’s not really happening in the healthcare sector. She doesn’t think that’s necessary either. It’s mainly the young freelancers, someone who has just started a business and has invested heavily and then suddenly can’t open his store. They have more need for it. She’s more like, ‘We’re just doing our job’. (T1BEGM09)

So, I hope that this [clapping] is something that is going to be long-lasting and I hope that it is something that has a positive consequence for those people [e.g., care professionals] as well. If only in a pay rise or extra leave this year. All of that will cost some money too but […] I’m just very scared of how quickly we’re going to be forgetting about this after the pandemic. How quickly people forget. How quickly are we going to want to go back to wanting to travel […]. (T1BEGM04)

The worry about forgetting what emerged as important or relevant for society in the pandemic is a concern that respondents reflected on more deeply, especially in the second round of interviews we conducted.

[In the beginning] there were these efforts, we go shopping for you etc., there were many incentives from the civilian population. Where one felt that this is an act of moving closer together—there was focus on the at-risk groups […]. And then, when the first restrictions were eased, it [the sense of togetherness] was, at least in my perception […] gone quite quickly. The comfort was back in focus, this idea of everyone for themselves, […] I think that nothing will change in a sustainable way, because one is spoilt with consumerism and with luxuries, and simply every person…one doesn’t want to give up the things that are comfortable. (T2ATMP07)

These concerns about the sustainability of the lessons learnt from the lockdowns exposed a set of nuanced understandings of institutional solidarity, sometimes expressed explicitly and sometimes implicitly. Explicit perceptions of, and expectations for, institutional solidarity included respondents discussing that individual acts of solidarity typically have relatively small impact compared with more institutionalised forms, such as an universal basic income or other forms of state social protection:

Maybe the neighbours will get a little closer, at least in my small environment, but in Spain this unconditional basic income has already been introduced, which would be good, of course, if several countries could get involved in trying it out, which I would find very good in the current situation. But I think that this applause for the doctors and nurses, that is, I’m afraid it won’t last long. (T1ATKK02)

Some interviewees expressed gratitude for state measures that provided safety nets to individuals and businesses at the beginning of the pandemic, and expected that this would lead to less societal disruption in the long-term.

I know people, especially in the restaurant industry, who have benefited from them [the government measures]. Either they are partially unemployed, so they can continue to pay their rent. On the other hand, I know other businesses that are not considered to be of primary importance, like this student who had an event-organising business that didn’t get anything, but, on the whole, I think people are being helped, there’s a net, there’s a safety net. If only by the fact that we have social security, we have health care. Solidarity, all this will mean that compared to other more liberal countries, there will be less breakage. (T1FRTS03)

The above excerpt underlines the perceived importance of institutionalised solidarity in the form of safety nets provided by the state in times of crisis and beyond. This was often accompanied by a strong sense of worry that an easing of restrictive measures and the gradual return to prepandemic life would open up a vacuum where solidaristic practices had been prominent during the pandemic.

People want to generate even more sales and accumulate even more. [They want] to have even more luxury goods for themselves, so that they really have something in bad times. I don’t think that solidarity will survive. What every individual can do, yes, okay, like me now in our house: we have a lot of elderly people here. But I was in touch with them before and I’m the kind of person who likes to approach people. I’ve baked, we’ve cooked for the people here. We’ve offered to shop for people. But the thing with the corporates or the rich, that is going to get worse. Much, much worse. They’re going to have more turnover, they’re going to have more Euros, they’re going to have more Bitcoins, they’re going to have more money in the banks, they’re going to do more sleazy deals so they can get more money. (T1CHBZ20)

This concern about the unsustainability of the kinds of solidarity seen during the pandemic was visible in both rounds of interviews, and in all countries. In some countries, such as Austria and Germany, respondents were explicit about what they thought was needed to hold on to some of the positive examples of societal cohesion during the pandemic, that is, more institutionalised policies of solidarity such as universal basic incomes. Respondents in other countries were less explicit about such policies, but reflected on the fact that collective solidarity would not last if big corporations, for example, are not held to higher standards and made to contribute to a more equal distribution of economic benefits and burdens in society. Institutionalised solidarity was seen as a crucial factor in mitigating against the multiple negative effects of the pandemic. Beyond the crisis, it was seen as an instrument to learn from and to develop policies that contribute to a stronger sense of solidarity in society while preventing a return to the prepandemic states of affairs.

## Discussion

A practice-focused understanding of solidarity allowed us to explore how solidarity was enacted—or not—at the individual and interpersonal level. Overall, there was a striking resonance across countries of a great deal of varied solidaristic practices in T1 (April and May 2020) with a demonstrable onset of ‘solidaristic fatigue’ in T2 (October 2020). Our findings show that adhering to measures was seen as a matter of solidarity as long as measures were viewed as effective and fair; when people felt that solidarity was needed but did not yet exist, they enacted it in different ways at a person-to-person level.

The abundance and variety of interpersonal and group-based examples of solidarity that emerged in the early weeks of the pandemic was striking. Our data show that the beginning of the pandemic provided space for extraordinary acts of solidarity, which in turn became moments in which people experienced solidarity and support in deep and sometimes challenging ways (eg, when lockdown rules were broken to assist others). In some cases, these experiences translated into a longing for solidaristic practices to last beyond the pandemic, with some respondents believing that this is only possible if governments step in to institutionalise policies that aid solidarity.

The solidaristic practices at the person-to-person and group-based level filled an important institutional void in a situation where formal support systems such as pandemic social security payments or support for local shops to set up an online shopping and delivery presence were yet to be devised. Our respondents seemed to have a shared understanding that individual solidaristic acts matter. In fact, when asked about their reasons for engaging in solidaristic practices, many offered deep reflections about what it means to be a member of society who is aware of the consequences of her actions or inactions, and who cares about the people around her. Such a tacit shared understanding may at least partly be due to the fact that the countries included in this study all have solidaristic healthcare and social security systems, which may have fostered an implicit sense of solidarity. As mentioned in the beginning, frequent appeals on the part of politicians and other public figures to citizens’ solidarity were more overt reminders of the indispensability of solidaristic action in a crisis.

Forms of assistance such as emotional and mental health support to people suffering from isolation, financial aid, donation and/or delivery of primary goods to those in need, are practices that emerged on individual (tier 1) or smaller organisations’ (tier 2) initiative, rather than as response to institutional calls. These practices often emerged to respond to a lack of institutional support practices (tier 3 solidarity), which were sometimes explicitly criticised as insufficient or inadequate. In this sense, the findings contribute to the medical humanities’ long-standing concern with ‘[…] influencing […] practitioners to refine and complexify their judgements […] in clinical situations, based on a deep and complex understanding […] of illness, suffering, personhood, and related issues’ (Shapiro et al. 2009, 192–193) by providing insights into human experiences of the pandemic, and how people view themselves in relationship to others, the healthcare system and the state. Our research illustrates that individual notions of solidarity can contextually and contingently lead to action, inaction or feelings of discontent, especially when other people’s actions are perceived as unsolidaristic.

This nuanced picture of solidarity can provide a useful starting point for future research, transitions and teachings in the medical humanities and beyond.

If we acknowledge that solidarity is needed for resilience in a public health crisis, then we can say that for it to endure it requires institutional support. As West-Oram (2021, 67) argues, ‘[e]ffective public health programmes cannot rely solely on private individuals always engaging in interpersonal solidarity in an optimal fashion’, they also need institutionalised solidarity— in the sense of strong healthcare systems and other institutions and policies that satisfy people’s basic needs and support the well-being of people and communities. Placing emphasis—and focusing financial and other resources—on institutions and practices of support for people who are disadvantaged would help to reduce inequalities and thus support social cohesion. It would also, we argue, bolster people’s ability and willingness to enact solidarity with others. Importantly, it would help to reverse some of the loss of trust that governments in many countries have caused by demanding solidarity of citizens while engaging in unsolidaristic actions themselves, such as vaccine nationalism, or protecting industry interests over the needs of workers. According to West-Oram (2021, 68), a ‘government which fails to engage in solidarity with its constituents, makes an implicit statement about the nature of the relationship between itself and the rest of society’.

In light of this, we strongly encourage public health and government officials to institutionalise practices of solidarity more systematically. Such manifestations and policies of solidarity might include, but are not limited to, policies introducing a universal basic income, strategies to award and recognise the hard work of healthcare workers in the pandemic either in monetary or structural terms or sharing resources such as vaccines with countries with less access (Geiger and Gross 2023; Geiger and McMahon 2021).

The opportunities for solidifying new forms of solidarity at the institutional level are numerous, but, as the definition of solidarity by Prainsack and Buyx (2017) suggests, solidarity comes at a cost; whether or not new inroads in the pursuit of institutionalised solidarity will be made therefore depends on policymakers’ willingness to accept the costs, for example, the costs associated with sharing healthcare resources with other countries, and to explain the need for such costs to society at large. Certainly, the data presented in this article suggest that many of the people we interviewed are willing to act in solidarity and that they are ready and even eager to see more political and social transformation as a result of the COVID-19 crisis.

Based on the interview data, we contend that there are several risks of not taking seriously the longing for more institutionalised forms of solidarity. For one, there is a risk that solidarity becomes tokenistic; if encouraged by officials, individuals may consider tokenistic practices such as clapping as an ‘easy’ or a less tiresome and costly substitute to other and arguably more impactful acts of solidarity. Collectively, these may also be seen as a tokenistic substitute for more decisive government action, for instance, paying healthcare workers more.

A second risk is that people’s sense of community may erode over time; we saw this in our data to a certain extent in the second phase of our interviews (in October 2020) where the former sense of ‘being in this together’ was partly replaced by a recognition of societal divisions, for instance, between ‘masked’ and ‘unmasked’ individuals (Schönweitz et al. 2022). Such divisions may especially affect those individuals who were particularly solidaristic in their practices and/or who carried greater costs of the pandemic. It is also vital at those stages of pandemic It is also vital at those stages of pandemic management where public health measures are being relaxed; institutionalised community-level solidarity scaffolds may in those cases be a bridge between state-level measures and an individualistic rhetoric of personal responsibility. For instance, community-level outdoor sports programmes or subsidised online grocery shopping may just be two such measures that would allow people to continue to adhere to safety measures once public health ones are relaxed.

We offer a number of observations from our research on why the medical humanities play a crucial role in addressing complex problems in health crises, and how their methodological approaches may be adapted in light of specific challenges of pandemics such as lockdowns. One of the core ambitions of the medical humanities is to shed light on the often overlooked lived experience of health and illness. The very nature of a pandemic means that the number of people affected by the experience of disease and crisis increases exponentially as anyone, and anywhere, can or will be affected. Methodological approaches need to be adjusted accordingly. We accounted for this specific feature of a pandemic health crisis by pursuing a large-scale, comparative, qualitative approach that embedded the study in transnational contexts. Additionally, our study provides ground for calls to offer a more integrated disciplinary approach that links the humanities and social sciences (Whitehead et al. 2016). The study illustrates what such an integrated disciplinary approach can look like and underlines the fruits that transdisciplinary and transboundary research collaboration can carry (Zimmermann et al. 2022).

Last but not least, we argue that the medical humanities have much to gain from directing their attention to individual health issues, and to health or illness situations that are experienced at a collective level, thus harnessing the previously mentioned emphasis on societal relationships in the humanities and social sciences (Green and Cladi 2020; Pickersgill and Smith 2021). The analysis of patient experiences through a collective lens such as the solidarity approach offers unique insights to understandings of the individual self and the collective community in a way that sheds light on health and social inequalities. By focusing on solidarity and solidarity practice, we have shown how the medical humanities might adapt to address poignant social justice issues without losing the ambition of uncovering the individual experience of disease, crisis and vulnerability.

We propose three advances which, in our view, are essential for research endeavours in the medical humanities that aim to map out existing social justice issues, and help uncover collective experiences of diseases and health crises: (1) a strong position for empirical and practice-based research alongside more normative approaches; (2) the confidence to make recommendations for practice and policymaking alike and (3) the pursuit of cross-national and multidisciplinary research collaborations in order to provide context to empirical findings and conceptual understandings of the situated nuances of social justice issues.

Given the size of the SolPan research consortium, we cannot emphasise this last point enough: the challenge of analysing data from nine countries was fruitfully addressed by a continuous, open and trustworthy exchange between team members, leaders and the whole consortium. With 40+ participating academics at different stages of their careers, we were able to draw on a vast array of skills, knowledge and training in different disciplines of the humanities and social sciences (eg, philosophy, bioethics, political science, anthropology, sociology), thus making it possible to probe our data from different angles.

## Limitations

While our cross-national, in-depth and longitudinal research bears crucial academic and policy insights, it also exhibits limitations. Although considerable at close to 700 interviews spread across a range of sociodemographics and 9 different countries, our research cannot be considered representative of the citizens of any particular nation or group of nations. Likewise, because of its non-random sample design it cannot be generalised. The qualitative design afforded us the opportunity to probe into people’s less obvious views and less overt practices more so than broader survey designs were able to. A second limitation is that while authors had on-the-ground exposure to public health messages and media discourses as they lived in the countries in which they did the research, for the purposes of this paper and due to the lens adopted we did not conduct a full public discourse analysis, which may have given further insights into tier 3 solidarity. A final limitation of the current article is that while we were sensitive to variations in our findings across countries, we did not engage in a systematic cross-country comparison.

## Conclusion

This article demonstrates that a practice-based understanding of solidarity can help shed light on a frequently used, but often poorly defined, concept. Practices of solidarity during the COVID-19 pandemic were abundant in our sample from nine European countries. Many respondents across all investigated countries told us that they felt the pandemic had exacer-bated previously existing health and social inequalities and that governments were not doing enough to mitigate these developments. Specific societal groups were highlighted as being particularly vulnerable to the negative knock-on effects of isolation and stay-at-home measures, namely people who live alone, children who cannot go to childcare or schools, healthcare workers who have to put themselves and their families at risk without adequate remuneration or recognition, the unemployed or self-employed and people who cannot work from home due to the nature of their work, to name but a few. The interview guide and the conceptual lens of solidarity provided a useful means for us to map out these social justice issues, and they brought to light a longing for more institutionalised forms of solidarity.

The study suggests that the medical humanities continue to play an important role, and that they can (and should) take on a more prominent role in uncovering collective experiences of disease or health crises. The pandemic reminded people, policy-makers and scientists alike that crises of large societal relevance can only be addressed when everybody contributes their part—at least in democratic countries. Investigating people’s lived experiences through the concept of solidarity sheds a light on the motivations and limits of this endeavour.

We offer three take-away messages for public health practitioners and policymakers: (1) solidarity can be an important motivating factor for compliance as well as non-compliance to public health measures, that is, to say that a nuanced picture about compliance emerges in which people sometimes disregard public health measures in order to act solidaristically towards particularly vulnerable groups. Compliance and solidarity are thus not equivalent (Spahl, Pot, and Paul 2022; Zimmermann et al. 2021); (2) different forms of solidarity, at different tiers, emerge during a public health crisis; it is important that public health officials and policymakers recognise and, where possible, support these forms of solidarity rather than leave them unrecognised or, even worse, act against them by, for example, pursuing policies that are overly (and overtly) focused on personal rather than collective responsibility; (3) the longing for institutionalised solidarity needs to be taken seriously and transformed into concrete institutional manifestations of solidarity. Our respondents expressed a strong wish not to return to life as it was pre-COVID-19, but rather think more sensibly and sustainably about the activities, actions and futures of societies.

## Supplementary Material

Supplementary Data

## Data Availability

Data are available on reasonable request. Data are stored in a secure location. All members of the consortium have access to the data. Access to the data is restricted to the members of the consortium to ensure the confidentiality and anonymity of the research participants.

## NOTE

1 The Italian country team carried out an additional round of interviews in June 2021. This quote is taken from this round of interviews, but the participant refers to the lockdowns that happened between March and October 2020, which is why the quotation is included here.
