# Supplementary material for: Solidarity during the COVID-19 pandemic: evidence from a nine-country interview study in Europe
Source: Med Humanit. Author manuscript; Available in PMC 2024 Sep 26. (PMC7616635; doi:10.1136/medhum-2022-012536)
Supplement: Supplementary Data [file EMS198470-supplement-Supplementary_Data.pdf]

| | Austria | | Belgium | | France | | Germany | | Ireland | | Italy | | The Netherlands | | Switzerland | | United Kingdom | |
|---|---|---|---|---|---|---|---|---|---|---|---|---|---|---|---|---|---|---|
| | **T1** | **T2** | **T1** | **T2** | **T1** | **T2** | **T1** | **T2** | **T1** | **T2** | **T1** | **T2** | **T1** | **T2** | **T1** | **T2** | **T1** | **T2** |
| **Total no of interviews (n)** | 80 | 72 | 36 | 22 | 22 | 21 | 47 | 43 | 32 | 25 | 33 | 29 | 32 | 29 | 31 | 25 | 35 | 30 |
| **Age** | | | | | | | | | | | | | | | | | | |
| 18-30 | 14 (18%) | 13 (18%) | 16 (44%) | 10 (45%) | 3 (14%) | 3 (14%) | 10 (21%) | 7 (16%) | 5 (16%) | 1 (4%) | 3 (9%) | 3 (10%) | 8 (25%) | 7 (24%) | 8 (26%) | 5 (20%) | 6 (17%) | 3 (10%) |
| 31-45 | 16 (20%) | 15 (21%) | 6 (17%) | 4 (18%) | 8 (36%) | 7 (33%) | 19 (40%) | 18 (42%) | 13 (41%) | 12 (48%) | 15 (45%) | 11 (38%) | 4 (13%) | 4 (14%) | 6 (19%) | 5 (20%) | 11 (31%) | 10 (33%) |
| 46-60 | 24 (30%) | 22 (31%) | 9 (25%) | 6 (27%) | 6 (27%) | 6 (29%) | 5 (11%) | 5 (12%) | 8 (25%) | 6 (24%) | 8 (24%) | 8 (28%) | 5 (16%) | 4 (14%) | 7 (23%) | 7 (28%) | 11 (31%) | 10 (33%) |
| 61-70 | 20 (25%) | 17 (24%) | 3 (8%) | 1 (5%) | 3 (14%) | 3 (14%) | 8 (17%) | 8 (19%) | 2 (6%) | 2 (8%) | 3 (9%) | 3 (10%) | 8 (25%) | 7 (24%) | 5 (16%) | 4 (16%) | 5 (14%) | 5 (17%) |
| 70+ | 6 (8%) | 5 (7%) | 2 (6%) | 1 (5%) | 2 (9%) | 2 (10%) | 5 (11%) | 5 (12%) | 4 (13%) | 4 (16%) | 4 (12%) | 4 (14%) | 7 (22%) | 7 (24%) | 5 (16%) | 4 (16%) | 2 (6%) | 2 (7%) |
| **Gender** | | | | | | | | | | | | | | | | | | |
| Female | 44 (55%) | 41 (57%) | 22 (61%) | 13 (59%) | 10 (45%) | 9 (43%) | 25 (53%) | 23 (53%) | 20 (63%) | 17 (68%) | 22 (67%) | 21 (72%) | 19 (59%) | 17 (59%) | 16 (52%) | 13 (52%) | 20 (57%) | 18 (60%) |
| Male | 36 (45%) | 31 (43%) | 14 (39%) | 9 (41%) | 12 (55%) | 12 (57%) | 22 (47%) | 20 (47%) | 12 (38%) | 8 (32%) | 11 (33%) | 8 (28%) | 13 (41%) | 12 (41%) | 15 (48%) | 12 (48%) | 14 (40%) | 11 (37%) |
| Other | 0 (0%) | 0 (0%) | 0 (0%) | 0 (0%) | 0 (0%) | 0 (0%) | 0 (0%) | 0 (0%) | 0 (0%) | 0 (0%) | 0 (0%) | 0 (0%) | 0 (0%) | 0 (0%) | 0 (0%) | 0 (0%) | 1 (3%) | 1 (3%) |
| **Household** | | | | | | | | | | | | | | | | | | |
| Single | 20 (25%) | 19 (26%) | 4 (11%) | 2 (9%) | 6 (27%) | 5 (24%) | 14 (30%) | 13 (30%) | 9 (28%) | 6 (24%) | 7 (21%) | 6 (21%) | 7 (22%) | 7 (24%) | 8 (26%) | 6 (24%) | 4 (11%) | 4 (13%) |
| Couple | 35 (44%) | 31 (43%) | 15 (42%) | 10 (45%) | 6 (27%) | 6 (29%) | 16 (34%) | 15 (35%) | 11 (34%) | 11 (44%) | 8 (24%) | 8 (28%) | 16 (50%) | 15 (52%) | 10 (32%) | 9 (36%) | 13 (37%) | 13 (43%) |
| Living with child(ren) < 12 | 8 (10%) | 7 (10%) | 3 (8%) | 2 (9%) | 6 (27%) | 6 (29%) | 8 (17%) | 7 (16%) | 5 (16%) | 4 (16%) | 6 (18%) | 5 (17%) | 3 (9%) | 3 (10%) | 3 (10%) | 2 (8%) | 8 (23%) | 8 (27%) |
| Living with child(ren) 12+ | 11 (14%) | 10 (14%) | 12 (33%) | 7 (32%) | 2 (9%) | 2 (10%) | 4 (9%) | 4 (9%) | 6 (19%) | 4 (16%) | 5 (15%) | 4 (14%) | 3 (9%) | 2 (7%) | 5 (16%) | 5 (20%) | 4 (11%) | 3 (10%) |
| Other | 6 (8%) | 5 (7%) | 2 (6%) | 1 (5%) | 2 (9%) | 2 (10%) | 5 (11%) | 4 (9%) | 1 (3%) | 0 (0%) | 7 (21%) | 6 (21%) | 3 (9%) | 2 (7%) | 5 (16%) | 3 (12%) | 6 (17%) | 2 (7%) |
| **Rural/urban** | | | | | | | | | | | | | | | | | | |
| Big town (e.g. capital, +500k) | 43 (54%) | 38 (53%) | 6 (17%) | 4 (18%) | 7 (32%) | 7 (33%) | 23 (49%) | 22 (51%) | 17 (53%) | 12 (48%) | 14 (42%) | 12 (41%) | 7 (22%) | 6 (21%) | 10 (32%) | 8 (32%) | 5 (14%) | 5 (17%) |
| Medium/small town | 19 (24%) | 17 (24%) | 15 (42%) | 7 (32%) | 9 (41%) | 9 (43%) | 12 (26%) | 11 (26%) | 10 (31%) | 9 (36%) | 11 (33%) | 9 (31%) | 15 (47%) | 13 (45%) | 6 (19%) | 5 (20%) | 18 (51%) | 15 (50%) |
| Rural (e.g. village) | 18 (23%) | 17 (24%) | 15 (42%) | 11 (50%) | 6 (27%) | 5 (24%) | 12 (26%) | 10 (23%) | 5 (16%) | 4 (16%) | 8 (24%) | 8 (28%) | 10 (31%) | 10 (34%) | 15 (48%) | 12 (48%) | 12 (34%) | 10 (33%) |
| **Employment status** | | | | | | | | | | | | | | | | | | |
| Employed (long-term contract) | 30 (38%) | 27 (38%) | 19 (53%) | 11 (50%) | 11 (50%) | 11 (52%) | 21 (45%) | 21 (49%) | 16 (50%) | 13 (52%) | 10 (30%) | 9 (31%) | 9 (28%) | 9 (31%) | 13 (42%) | 11 (44%) | 17 (49%) | 15 (50%) |
| Self-employed | 15 (19%) | 14 (19%) | 4 (11%) | 3 (14%) | 1 (5%) | 1 (5%) | 4 (9%) | 4 (9%) | 4 (13%) | 4 (16%) | 9 (27%) | 8 (28%) | 4 (13%) | 3 (10%) | 3 (10%) | 3 (12%) | 5 (14%) | 3 (10%) |
| Employed (short-term/precarious contract) | 5 (6%) | 3 (4%) | 3 (8%) | 2 (9%) | 3 (14%) | 3 (14%) | 4 (9%) | 3 (7%) | 2 (6%) | 2 (8%) | 3 (9%) | 3 (10%) | 5 (16%) | 4 (14%) | 6 (19%) | 4 (16%) | 2 (6%) | 2 (7%) |
| Unemployed | 5 (6%) | 5 (7%) | 3 (8%) | 3 (14%) | 0 (0%) | 0 (0%) | 4 (9%) | 3 (7%) | 2 (6%) | 1 (4%) | 2 (6%) | 2 (7%) | 4 (13%) | 3 (10%) | 1 (3%) | 1 (4%) | 4 (11%) | 3 (10%) |
| Retired | 19 (24%) | 17 (24%) | 5 (14%) | 2 (9%) | 4 (18%) | 4 (19%) | 10 (21%) | 10 (23%) | 4 (13%) | 4 (16%) | 3 (9%) | 3 (10%) | 10 (31%) | 10 (34%) | 7 (23%) | 6 (24%) | 5 (14%) | 5 (17%) |
| Other | 6 (8%) | 6 (8%) | 2 (6%) | 1 (5%) | 3 (14%) | 2 (10%) | 4 (9%) | 2 (5%) | 4 (13%) | 1 (4%) | 6 (18%) | 4 (14%) | 0 (0%) | 0 (0%) | 1 (3%) | 0 (0%) | 2 (6%) | 2 (7%) |
| **Education** | | | | | | | | | | | | | | | | | | |
| Less than 10 years | 8 (10%) | 7 (10%) | 1 (3%) | 0 (0%) | 2 (9%) | 2 (10%) | 2 (4%) | 2 (5%) | 2 (6%) | 2 (8%) | 2 (6%) | 2 (7%) | 3 (9%) | 2 (7%) | 10 (32%) | 7 (28%) | 2 (6%) | 2 (7%) |
| 10-14 years (e.g. highschool diploma) | 27 (34%) | 23 (32%) | 10 (28%) | 7 (32%) | 6 (27%) | 6 (29%) | 16 (34%) | 14 (33%) | 3 (9%) | 2 (8%) | 17 (52%) | 16 (55%) | 13 (41%) | 11 (38%) | 3 (10%) | 2 (8%) | 10 (29%) | 6 (20%) |
| Higher education | 45 (56%) | 42 (58%) | 25 (69%) | 15 (68%) | 14 (64%) | 13 (62%) | 29 (62%) | 27 (63%) | 27 (84%) | 21 (84%) | 14 (42%) | 11 (38%) | 16 (50%) | 16 (55%) | 18 (58%) | 16 (64%) | 23 (66%) | 22 (73%) |
| **Household net income prior to pandemic** | | | | | | | | | | | | | | | | | | |
| Up to 1400€/1200GBP/ 4000CHF per month | 9 (11%) | 9 (13%) | 3 (8%) | 2 (9%) | 3 (14%) | 3 (14%) | 6 (13%) | 2 (5%) | 3 (9%) | 1 (4%) | 5 (15%) | 4 (14%) | 7 (22%) | 5 (17%) | 6 (19%) | 5 (20%) | 5 (14%) | 2 (7%) |
| 1401€/1201GBP/4001CHF - 3000€/2600GBP/7000CHF per month | 29 (36%) | 27 (38%) | 18 (50%) | 11 (50%) | 3 (14%) | 3 (14%) | 14 (30%) | 15 (35%) | 9 (28%) | 7 (28%) | 22 (67%) | 19 (66%) | 14 (44%) | 13 (45%) | 9 (29%) | 7 (28%) | 5 (14%) | 5 (17%) |
| More than 3000€/2600GBP/ 7000CHF per month | 42 (53%) | 36 (50%) | 15 (42%) | 9 (41%) | 16 (73%) | 15 (71%) | 27 (57%) | 26 (60%) | 20 (63%) | 17 (68%) | 6 (18%) | 6 (21%) | 11 (34%) | 11 (38%) | 16 (52%) | 13 (52%) | 25 (71%) | 23 (77%) |
